# Influence of the Postovulatory Progesterone on the Outcome of In Vitro Fertilization

**DOI:** 10.7759/cureus.104495

**Published:** 2026-03-01

**Authors:** Emina Ejubovic, Miro Kasum, Emir Mahmutbegovic, Jasmin Hodzic, Emina Sacak

**Affiliations:** 1 Department of Gynaecology, Obstetrics and Reproductive Endocrinology, Sarajevo Medical School, Sarajevo School of Science and Technology, Sarajevo, BIH; 2 Department of Obstetrics and Gynaecology, University Hospital Centre Zagreb, University of Zagreb, Zagreb, HRV; 3 Department of Gynecology, Obstetrics and Reproductive Endocrinology, Sarajevo Medical School, Sarajevo School of Science and Technology, Sarajevo, BIH; 4 Department of Gynaecology and Obstetrics, Faculty of Medicine, University of Zenica, Zenica, BIH; 5 Department of Internal Medicine, Zenica Cantonal Hospital, Zenica, BIH

**Keywords:** in vitro fertilization, ivf, luteal support, postovulatory progesterone, s: preovulatory progesterone

## Abstract

Aim: To investigate the influence of postovulatory progesterone (P4) on the outcome of in vitro fertilization (IVF).

Materials and methods: This was a prospective cohort study which included 400 patients (300 in the control group and 100 in the group with elevated preovulatory P4 (preovulatory P4 > 4.77 nmol/L) - 85 normal responders and 15 high responders). The influence of postovulatory P4 on the IVF outcome has been investigated in this study.

Results: It was found that the P4 value on the fifth cycle day at a concentration ≤425.7 nmol/L has a high sensitivity (86.21%, 95% CI 81.7-90.0%) and a low specificity (23.64%, 95% CI 16.1-32.7%) in all patients in the study. The obtained results showed that P4 values on day 5 >425,7 nmol/L in all patients, and the P4 on the 5th day >384,6 nmol/L among patients with elevated preovulatory P4 represent a threshold for the successful IVF procedure (positive ultrasound visualization of the gestational sac at six weeks of pregnancy with the presence of fetal heart action after embryo transfer).

Conclusion: The P4 on the fifth day >425,7 nmol/L could be a potential marker for the success of the IVF procedures among all the patients, and with P4 on the fifth day >384,6 nmol/L among patients with elevated preovulatory P4.

## Introduction

Research on the influence of postovulatory progesterone (P4) or early luteal serum P4 on day five, under standardized luteal support, on the outcome of the IVF procedure has been scarce so far. One group of authors believes that the outcome of pregnancies in in vitro fertilization (IVF) procedures is better if estradiol (E2) is relatively elevated and P4 values are high [1], while another group of authors believes that intense periovulatory maturation of the endometrium adversely affects the outcome of IVF procedures [2]. There are numerous controversies in the literature regarding the value of postovulatory P4. The mean value of P4 determined after ovulation on day five (day of blastocyst transfer) varies, depending on the study, from 150 nmol/L to even 315 nmol/L with the support of corpus luteum with micronized P4 [3,4]. If P4 was determined on day three (day of embryo transfer), its mean value was 85 nmol/L, also with progesterone support of the corpus luteum [5]. The problem of postovulatory P4 has been actualized during the last few years. Various studied have attempted to show whether serum P4 values during the mid-luteal phase can serve as a predictive factor for the outcome of the IVF procedure and whether increasing the dose of exogenous P4 in patients with low values during the mid-luteal phase can improve the pregnancy rate [6]. The degree of luteolysis expressed through P4 values 48 hours after the oocyte aspiration was the subject of a recent study in which the gonadotropin-releasing hormone antagonist (GnRH-ant) protocol was used, and the final maturation of oocytes was achieved using GnRH. The degree of luteolysis was shown to vary widely among patients with P4 values from 13.0 ng/mL (41.3 nmol/L) to ≥60.0 ng/mL (190.8 nmol/L) measured 48 hours after oocyte aspiration station [7]. Another study has shown that the final maturation of the oocytes with the use of GnRH causes expressed luteolysis that cannot be compensated by the standard luteal support with progesterone, and therefore high doses of P4 or hCG are needed to prevent the luteolysis. However, it has also been shown that luteolysis is not always complete after the use of GnRH for the final maturation. In high responder patients (number of follicles >20 on hCG day, E2>3000 pg/mL), the evaluation of E2 and P4 values in the serum 48 hours after the oocyte aspiration has shown that luteolysis with GnRH agonists (GnRH-a) trigger is individually specific, even in high responders with the same number of oocytes [8]. Another study tried to find a connection between the value of postovulatory P4 and a successful IVF cycle. It was shown that a P4 value of 17 ng/mL (54 nmol/L) measured on the 7th day after the embryo transfer is a better predictor of a successful IVF procedure, but that increasing the dose of progesterone to 300mg per day as a luteal support had no effect on a successful outcome [6].

The main goal of this paper is to investigate whether the postovulatory progesterone on day five predicts a successful IVF procedure in fresh and frozen thawed embryo transfer, presented as a positive ultrasound visualization of the gestational sac at six weeks of pregnancy, with the presence of fetal heart action after embryo transfer, and to determine ROC-based cutoffs overall and stratified by preovulatory P4 status.

## Materials and methods

This prospective cohort study was conducted at the Clinic for Women’s Diseases and Childbirth, Petrova, Zagreb, Croatia, between January 1st, 2015 and December 31st, 2017. A total of 400 patients undergoing in vitro fertilization (IVF) were included in the study. Patients were excluded if they met any of the following criteria: elevated progesterone (P4) levels on cycle days 2-4, age over 37 years, or poor ovarian response (defined as <4 follicles on the day of hCG administration and/or estradiol [E2] <500 pg/mL), as these parameters are known to negatively affect IVF outcomes. Additional exclusion criteria included intrauterine insemination (IUI), use of anti-estrogens (clomiphene citrate or letrozole) during IVF stimulation, and fertility preservation procedures performed due to malignant disease. All remaining patients undergoing standard IVF procedures were eligible for inclusion. Written informed consent was obtained from all participants after explanation of the potential adverse effects of elevated P4 levels on IVF outcomes. The study was conducted in accordance with institutional ethical standards and the Declaration of Helsinki (1975, revised 1983). The research has been approved by the Ethical committee University of Zagreb, School of Medicine, No 251-59-10106-25-111/42 from December 11, 2015.

Serum P4 and E2 levels were measured on the day of hCG administration. Patients with normal preovulatory P4 levels (<4.77 nmol/L) constituted the control group (n = 300), while patients with elevated P4 levels (>4.77 nmol/L) formed the study group (n = 100). The study group was further subdivided into: normal responders (n = 85): ≤15 follicles on the day of hCG and E2 <3,000 pg/mL, and high responders (n = 15): E2 ≥3,000 pg/mL and >20 oocytes retrieved. All groups were matched for age, body mass index (BMI), stimulation protocol, gonadotropin dosage, number of retrieved oocytes, and number of transferred embryos. The responder status has been used for the stratification of patients. Controlled ovarian stimulation was performed using gonadotropins in combination with gonadotropin-releasing hormone (GnRH) analogues. The choice and dosage of medication were individualized based on patient age, baseline follicle-stimulating hormone (FSH), antral follicle count (AFC), anti-Müllerian hormone (AMH), BMI, and prior IVF response.

Lower gonadotropin doses were used in younger patients with higher ovarian reserve. Patients with polycystic ovaries received predominantly FSH-based preparations with minimal luteinizing hormone (LH) activity. In cases with an increased risk of ovarian hyperstimulation syndrome (OHSS), mild stimulation protocols with GnRH antagonists and GnRH agonists for ovulation triggering were applied. The medications used included follitropin alfa (Gonal F®, Merck Serono, Germany), follitropin beta (Puregon®, Organon, The Netherlands), menotropin (Menopur®, Ferring Pharmaceuticals, Switzerland), triptorelin (Decapeptyl SR®, Ferring Pharmaceuticals, Switzerland), cetrorelix acetate (Cetrotide®, Merck Serono, Germany), and choriogonadotropin alfa (Ovitrelle®, Merck Serono, Germany).

Follicular development was monitored by transvaginal ultrasound and serum E2 measurements. Ultrasound examinations were performed using a Voluson S8 system (General Electric) with a GE RIC5-9W-RS 3D/4D vaginal probe (4-9 MHz). When at least three follicles measuring ≥17 mm in diameter were observed, hCG was administered. Transvaginal ultrasound-guided oocyte retrieval was performed 36 hours later. Procedures were carried out with patients fasting, under analgosedation (pethidine and diazepam) or local anesthesia. A 16-gauge needle (1.1 mm diameter) was used for follicular aspiration. Retrieved follicular fluid was immediately transferred to the embryology laboratory for assessment of oocyte number, maturity, and quality. Atrophic and degenerated oocytes were excluded from further procedures. Semen samples were analyzed and prepared in the laboratory, and fertilization was achieved using intracytoplasmic sperm injection (ICSI).

All patients received luteal phase support with vaginal micronized progesterone (Utrogestan®, Laboratories Besins International, France) at a daily dose of 600 mg (three doses of 200 mg) for 12 days. Given population-specific variations in postovulatory P4 levels, the mean serum P4 value on day 5 post-ovulation was calculated in the control group and used as a discriminatory threshold. Based on this value, patients in the study group were allocated to frozen-thawed embryo transfer (FET) or blastocyst embryo transfer (BET). In normal responders, FET was performed when day-5 P4 levels exceeded the discrimination limit, while BET was performed when P4 levels were below the threshold. In high responders, patients were randomized to either fresh or delayed blastocyst transfer. The primary objective of the study was to evaluate the influence of postovulatory progesterone levels on IVF outcomes.

## Results

An analysis of the influence of P4 on the fifth day on the outcome of IVF was made. It was found that the P4 value on day five at a concentration ≤425.7 nmol/L has a high sensitivity (86.21%, 95% CI 81.7-90.0%) and a low specificity (23.64%, 95% CI 16.1-32.7%) in all study patients in the IVF procedure; that is, with a P4 value on day five of ≤425.7 nmol/L, there is a high sensitivity and low specificity that the study patients will not have a positive IVF outcome (Table 1).

**Table 1:** Influence of P4 on the fifth day on the outcome of in vitro fertilization in all patients. AUC: Area under curve; SE: Standard error; CI: Confidence interval

AUC	0.545
SE	0.0330
95% CI	0.495 - 0.595
Z value	1.374
P value	0.1695

Analysis of P4 on day five suggests that P4 on day five at a concentration of ≤425.7 nmol/L is a good indicator for IVF procedure failure, but if failure occurs, P4 should not be considered responsible for the failed pregnancy. 

An analysis of the influence of P4 on the fifth day of conception rate was made in the control group. No statistically significant association was found between P4 concentration on day five of >193.4 nmol/L and conception rate (Table 2).

**Table 2:** Influence of P4 concentration on the fifth day on conception in the control group. AUC: Area under curve; SE: Standard error; CI: Confidence interval

AUC	0.515
SE	0.0356
95% CI	0.457 - 0.573
Z value	0.431
P value	0.667

An analysis was made of the influence of P4 on the fifth day on the clinical pregnancy rate among patients of the control group. In the control group, no statistically significant association between P4 concentration on day five of >193.4 nmol/L and clinical pregnancy rate was found (Table 3).

**Table 3:** Influence of P4 concentration on the fifth day on the clinical pregnancy rate in the control group AUC: Area under curve; SE: Standard error; CI: Confidence interval

AUC	0.501
SE	0.0378
95% CI	0.443 - 0.559
Z value	0.0137
P value	0.9890

In the study group, ROC analysis was performed to determine the influence of P4 concentration on the fifth day of the cycle on the clinical pregnancy rate. Table 4 shows the values ​​of the area under the curve (AUC) together with the values ​​of the statistical test. The best ratio of sensitivity and specificity of the test was measured in P4 concentration on day five, ≤384.6 nmol/L, showing modest discrimination; at that concentration, sensitivity was 71.9% and specificity 63.9%.

**Table 4:** Influence of P4 concentration on the fifth day on the conception rate in the study group. AUC: Area under curve; SE: Standard error; CI: Confidence interval

AUC	0.625
SE	0.061
95% CI	0.522 - 0.719
Z value	2.050
P value	0.040

Analyzing the clinical pregnancy rate and P4 values ​​on the fifth day in the study group, it was determined that 56.1% of cases with a positive clinical pregnancy rate at P4 values ​​on the fifth day >384.6 nmol/L compared to 22.0% of cases with a negative clinical pregnancy rate with a P4 value of 384.6 nmol/L on the 5th day; it represents a protective factor (RR=0.563) for the pregnancy rate, that is, the risk that the patient will miscarry is lower if the P4 on the fifth day is >384.6 nmol/L (Table 5).

**Table 5:** Relative risk of P4 concentration on the fifth day >384.6 nmol/L for the clinical pregnancy rate in the study group. χ 2 =12.182; P<0.001 RR=0.563; Z=3.029; P=0.003

			Clinical pregnancy rate		Total
			Positive	Negative	
P4, 5th day >384.6 nmol/L	No	N (%)	13 (22.0)	46 (78.0)	59 (59.0)
	Yes	N (%)	23 (56.1)	18 (43.9)	41 (41.0)

P4 on day five for clinical pregnancy rate in the study group did not have high sensitivity (69.12%, 95% CI 56.7-79.8%) and specificity (62.50%, 95% CI 43.7-78.9%) at a P4 value on day 5 of ≤384.6 nmol/L (Table 6).

**Table 6:** Influence of P4 concentration on the fifth day of the cycle on the clinical pregnancy rate in the study group. AUC: Area under curve; SE: Standard error; CI: Confidence interval

AUC	0.614
SE	0.0630
95% CI	0.511 - 0.710
Z value	1.809
P value	0.0704

## Discussion

The clinical significance of P4 during the luteal phase in predicting pregnancy outcome after controlled ovarian hyperstimulation (COH) is still inadequately investigated. It is known that the survival of the corpus luteum 7-8 days after oocyte retrieval can be an early diagnostic sign of pregnancy. Also, P4 is sometimes referred to as “pregnancy hormone” due to its role in implantation and maintenance of pregnancy. Research by Gumaa et al. [3] showed significantly higher P4 values in the serum of pregnant patients compared to those patients in whom pregnancy was not registered, both in the early luteal phase and in the late one. The presumed mechanism of action of P4 during early pregnancy involves the preparation of the endometrium by means of E2 and the reduction of uterine smooth muscle contractility. These statements were confirmed by Fanchin in 2001 [9], who showed that uterine contractions on the day of embryo transfer have a negative effect on the outcome of the transfer itself. A negative correlation was also found between the frequency of uterine contractions and the value of P4 in the serum, thus emphasizing the importance of P4 in IVF procedures [3]. Brady et al. found a positive correlation of P4 values on the day of transfer with the clinical pregnancy rate and the live birth rate. Furthermore, they also showed that increasing the dose of P4 after the transfer was sufficient to improve the outcome [5]. Due to the limited number of studies regarding the influence of P4 on the 5th day on the outcome of the IVF procedure, we also analyzed this parameter in our research. It was shown that P4 on the 5th day demonstrated limited discriminatory performance for a successful IVF outcome (AUC 0.545, P=0.1695), with a high sensitivity (86.21%, 95% CI 81.7- 90.0%) and a low specificity (23.64%, 95% CI 16.1-32.7%) for the overall outcome of in vitro fertilization in all subjects at a P4 value on day 5 of ≤425.7 nmol/L suggesting that day-5 P4 alone is unlikely to serve as a robust standalone predictor. In clinical terms, this means that P4 on day five is a good marker for IVF failure, but if failure occurs, P4 should not be seen as responsible for the failed pregnancy. Namely, it is a well-known fact that, in addition to P4, numerous factors such as hormonal imbalance, coagulability disorders, infections and nutrition can affect the outcome of IVF [10-12], and that our results should also be viewed through this fact.

On the other hand, in the study group (P4>4.77 nmol/L), ROC analysis was performed to determine the predictive influence of P4 concentration on the fifth day of the cycle on the clinical pregnancy rate. The best ratio of sensitivity and specificity of the test was measured in P4 concentration on day five, ≤384.6 nmol/L. At that concentration, sensitivity was 71.9% and specificity 63.9% (P=0.040). Analyzing the subjects with a positive clinical pregnancy rate and P4 values on day five, it was determined that 56.1% of positive clinical pregnancy rates with P4 values on day 5 >384.6 nmol/L, as compared to 22.0% of negative clinical pregnancy rates with P4 values on day five of 384.6 nmol/L, which represents a protective factor (RR=0.563) of pregnancy, that is, the risk that the subject will have a negative clinical pregnancy rate is lower if P4 on day five is >384.6 nmol/L. High sensitivity (69.12%, 95% CI 56.7-79.8%) and specificity (62.50%, 95% CI 43.7-78.9%) were not shown regarding the influence of P4 on the fifth day from ≤384.6 nmol/L on the clinical pregnancy rate in the studied group. These findings suggest that, if we do not take into account the value of preovulatory P4 in the IVF procedure, P4 values on day five of >425.7 nmol/L may warrant evaluation in prospective trials. Similarly, if the patient has normal P4 values on the day of hCG, however, the postovulatory P4 value should be >384.6 nmol/L, given that we have proven that this value can be a protective factor for pregnancy. The results of our research are in accordance with the research of Franco Dias [13], Xu [14] and Racca [15]. These findings support further prospective evaluation of individualized luteal phase support strategies, which should be adjusted in the period after embryo transfer, with the aim of reducing the number of failed IVF attempts. both in patients with normal and in patients with elevated preovulatory P4 values. In other words, stimulation protocols and luteal support should be individualized for each patient.

Ultimately, when looking at day five P4 in subjects with normal preovulatory P4 values, one should aim for values >425.7 nmol/L as a potential marker of a successful clinical pregnancy rate and, in most cases, a better ongoing pregnancy rate. On the other hand, if we are talking about subjects with elevated preovulatory P4 values, the P4 value on the day of embryo transfer should be >384.6 nmol/L for a successful IVF procedure. Future research will show whether the aforementioned strategies improve the outcome of the IVF procedure with elevated values of the preovulatory P4 result, and to what extent the individualization of luteal support after embryo transfer has been successful.

## Conclusions

In the study group with elevated preovulatory P4, the pregnancy outcome was better in normal responders with higher concentrations of postovulatory P4 in whom, after cryopreservation/thawing, blastocyst transfer was performed in a natural cycle, compared to normal responders with lower concentrations of postovulatory P4 (BET). In the study group with randomized embryo transfer in high responders with elevated values ​​of preovulatory P4 and postovulatory P4, day five P4 shows limited predictive value overall, with modest discrimination in the elevated preovulatory P4 subgroup. The ongoing pregnancy rate is more pronounced in cases of transfer of fresh blastocysts in the same cycle (BET), in relation to embryo cryopreservation/thawing and transfer in the natural cycle.

P4 values ​​on the fifth day >425.7 nmol/L represent a potential marker of a higher conception rate, and in most cases, a higher clinical pregnancy rate at normal preovulatory P4 values ​. P4 values ​​on the fifth day >384.6 nmol/L represent a potential marker of a higher conception rate, and in most cases, a higher clinical pregnancy rate with elevated preovulatory P4 values. Interpretation is limited by potential confounding related to transfer strategy decisions guided by progesterone levels. Individualization of stimulation protocols and luteal support after embryo transfer should improve the outcome of IVF procedures.
